# The GimA Locus of Extraintestinal Pathogenic *E. coli*: Does Reductive Evolution Correlate with Habitat and Pathotype?

**DOI:** 10.1371/journal.pone.0010877

**Published:** 2010-05-28

**Authors:** Timo Homeier, Torsten Semmler, Lothar H. Wieler, Christa Ewers

**Affiliations:** 1 Institute for Microbiology and Epizootics, Veterinary Faculty, Free University Berlin, Berlin, Germany; 2 Institute of Animal Hygiene and Veterinary Public Health, Faculty of Veterinary Medicine, University of Leipzig, Leipzig, Germany; University of Würzburg, Germany

## Abstract

*IbeA* (invasion of brain endothelium), which is located on a genomic island termed GimA, is involved in the pathogenesis of several extraintestinal pathogenic *E. coli* (ExPEC) pathotypes, including newborn meningitic *E. coli* (NMEC) and avian pathogenic *E. coli* (APEC). To unravel the phylogeny of GimA and to investigate its island character, the putative insertion locus of GimA was determined via Long Range PCR and DNA-DNA hybridization in 410 *E. coli* isolates, including APEC, NMEC, uropathogenic (UPEC), septicemia-associated *E. coli* (SEPEC), and human and animal fecal isolates as well as in 72 strains of the *E. coli* reference (ECOR) collection. In addition to a complete GimA (∼20.3 kb) and a locus lacking GimA we found a third pattern containing a 342 bp remnant of GimA in this strain collection. The presence of GimA was almost exclusively detected in strains belonging to phylogenetic group B2. In addition, the complete GimA was significantly more frequent in APEC and NMEC strains while the GimA remnant showed a higher association with UPEC strains. A detailed analysis of the *ibeA* sequences revealed the phylogeny of this gene to be consistent with that obtained by Multi Locus Sequence Typing of the strains. Although common criteria for genomic islands are partially fulfilled, GimA rather seems to be an ancestral part of phylogenetic group B2, and it would therefore be more appropriate to term this genomic region GimA locus instead of genomic island. The existence of two other patterns reflects a genomic rearrangement in a reductive evolution-like manner.

## Introduction

The bacterial species *Escherichia coli* reflects a high degree of diversity, and includes commensal, extraintestinal pathogenic *E. coli* (ExPEC) and intestinal pathogenic strains [1]. Although not mutually exclusive, ExPEC, which predominantly belong to phylogenetic group B2, are currently categorized based on their original host and/or clinical background resulting in the designation of pathotypes newborn meningitis causing *E. coli* (NMEC), uropathogenic *E. coli* (UPEC), avian pathogenic *E. coli* (APEC), and septicemia-associated *E. coli* (SEPEC) [1], [2], [3].

NMEC are well known as causative agents of newborn meningitis, representing one of the five leading neonatal infections worldwide [4], [5]. Even countries with highly developed health care systems encounter high rates of mortality and morbidity due to the disease [6], [7]. The development of bacterial meningitis includes several pathogenic steps, involving mucosal colonization in the gastrointestinal tract, microbial translocation of the mucous membrane and invasion of the intravascular space with subsequent intravascular survival and accompanied bacteremia. After crossing the brain blood barrier and invading the central nervous system (CNS), both representing key steps in the pathogenesis of bacterial meningitis, inflammatory and toxic processes are induced, which finally lead to meningitis [8], [9]. Several factors have been reported to be involved in the invasion process, e.g. encoded by *aslA* [10], *ibeA* [11], [12], *ibeB* [13], *yijP* [14] and *ompA* [15]. While *aslA*, *ibeB*, *yijP* and *ompA* have homologues present in non pathogenic *E. coli* K-12 strains [10], [13], [14], [15], this is not the case for *ibeA* [11], [16], which has originally been identified in archetypical NMEC strain RS218 (O18:K1:H7; ST95) through a Tn*pho*A mutagenesis approach [12]. Subsequent analyses demonstrated that an *ibeA* knock-out mutant of this strain showed reduced invasion of human brain microvascular endothelial cells (HBMEC) and attenuated virulence in a newborn rat model [11]. There has also been evidence for an involvement of *ibeA* in the pathogenesis of systemic *E. coli* infections in chickens, as a knock-out mutant of APEC strain BEN2908 (O2:K1:H5; ST95) was attenuated *in vivo* in a chicken infection model [17].

Sequence analysis revealed *ibeA* to be part of a 20.3 kb gene cluster located between *yjiD* and *yjiE*, adjacent to the *fim* Operon [18]. Due to the overall G+C content of 46.2%, which differs significantly from that of the remaining RS218 chromosome (50.8%), it seemed reasonable to define this gene cluster as a genomic island, which was termed GimA (genomic island of newborn meningitis causing *E. coli* containing the invasion locus *ibeA*) [18]. GimA consists of 14 open reading frames, organized in four operons (GimA1: *ptnIPKC*, GimA2: *cglDTEC*, GimA3: *gcxKRCI* and GimA4: *ibeRAT*). The functions of the gene products were assigned to the categories of proteins related to substrate transportation and carbon source metabolism, the latter one particularly known to be involved in bacterial stress response [19].

While the role of GimA, and particularly of IbeA, in the pathogenesis of newborn meningitis is well characterized, its distribution in the phylogenetic background of the *E. coli* population is scarcely investigated. Several publications have described the occurrence of *ibeA* in ExPEC pathotypes, including NMEC (38.5–58.9% *ibeA* positive), UPEC (18.2–19.2% *ibeA* positive) and APEC (14.2–26.2% *ibeA* positive) [17], [20], [21], [22]. Although there was a notion that *ibeA* might be of predictive value for the presence of B2 strains, its overall irregular occurrence in this group of strains contradicted this assumption [20], [21], [23].

The present study was performed (i) to investigate the distribution of *ibeA* and GimA in association with the phylogenetic background of ExPEC strains as determined by PCR analyses and multi locus sequence typing, and (ii) to get an insight into the evolutionary origin of GimA by determining potential structural differences in the genetic composition of this putative island, as well as by analysis of the *ibeA* gene sequences of phylogenetically related strains. The resulting data should shed light on the fate of GimA in the evolution of *E. coli* after its supposed initial integration into the chromosome.

## Materials and Methods

### Bacterial strains, Multi locus sequence typing and DNA purification

A total of 410 *E. coli* strains, including 338 wild type strains and 72 strains from the ECOR collection [24] (Table S1) were investigated. In detail, the entire collection consisted of 98 APEC strains, isolated from septicemia in birds, 140 UPEC strains, implicated in urinary tract infections in humans (n = 64), cats (n = 22), and dogs (n = 54), 25 newborn meningitic *E. coli* (NMEC) strains, 28 SEPEC strains from cases of septicemia in humans, and 119 fecal strains from clinically healthy humans (n = 86) and animals (n = 33).

All strains have been assigned to multi locus sequence types (STs) according to the scheme described by Wirth et al. [25] using primers previously published [26]. New STs were submitted to the MLST database (http://mlst.ucc.ie/mlst/mlst/dbs/Ecoli). An ST complex (STC) has been defined to include at least three STs that differ from their nearest neighbour by no more than one of seven alleles. Some strains and their STs have already been published elsewhere [25], [27], [28], [29].

Genomic DNA was isolated using the MasterPure™ Genomic DNA Purification Kit (EPICENTRE Biotechnologies, Madison, WI, USA), following the manufacture's instructions and kept at 4°C until further use.

Additionally, publicly available whole genome sequences of *E. coli* strains were included: UPEC strains CFT073 (Acc. No AE014075) [30], UTI89 (Acc. No CP000243) [31], F11 (Acc. No AAJU00000000) [32], and 536 (Acc. No CP000247) [33]; APEC strain APEC O1 (Acc. No CP000468) [34] and K-12 strain MG1655 (Acc. No U00096) [35]. The GimA sequence of NMEC strain RS218 (Acc. No AF289032) [18] was also included in the study.

### Analysis of the GimA locus: PCR analyses

**Figure 1:**
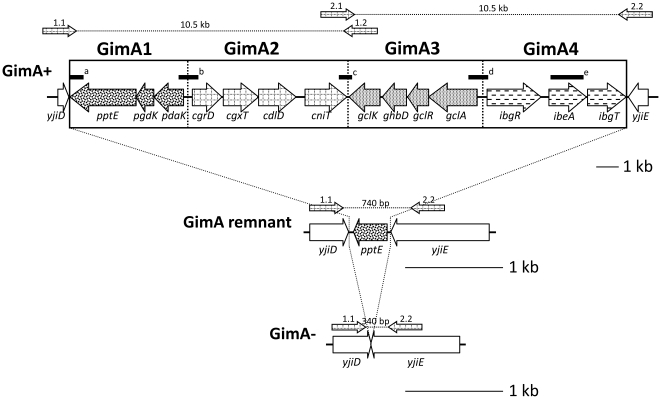
Schematic presentation of GimA locus patterns (GimA+, GimA remnant, GimA-), primer binding sites [1.1 (*yjiD-E* FP) and 1.2 (*gclKGimA3* RP), 2.1 (*cniTGimA2* FP) and 2.2 (*yjiD-E* RP)], and probe binding sites (black bars [a–e]).

In a first step to analyze the GimA locus we performed a PCR screening approach, using primers (MWG, Ebersberg, Germany) matching up- (*yjiD*) and downstream (*yjiE*) of the GimA locus (yjiD-yjiE FP and yjiD-yjiE RP), respectively (Figure 1, Table 1). The reaction mixture, containing a final volume of 25 µl, was prepared according to standard protocols [36]. PCR buffer, MgCl_2_, and Taq-Polymerase were purchased from Rapidozym, (Berlin, Germany). The samples were subjected to 25 cycles of amplification in a thermal cycler (TProfessional, Biometra, Goettingen, Germany). Reaction conditions were 10 min of initial denaturation at 94°C, followed by 25 cycles of denaturation (30 sec at 94°C), annealing (30 sec for 55°C) and elongation (60 sec at 72°C) and a final elongation at 72°C for 10 minutes. Due to the PCR conditions the maximum product size was limited to approximately 1.5 kb. Strains lacking an amplicon were subsequently analyzed with a long range PCR approach using the Extensor Hi-Fidelity PCR Master Mix (ABgene Germany, Hamburg, Germany), targeting two overlapping fragments of GimA by the use of primer pairs yjiD-yjiE FP/gclKGimA3 RP and cniTGGimA2 FP/yjiD-yjiE RP (Table 1) as demonstrated in Figure 1.

**Table 1:** Oligonucleotide primers used for the characterization of the GimA locus of *E. coli* strains: sequences, target regions, melting temperatures and amplicon sizes for each primer set and application in the present study.

Application	Primer name	Sequence 5′-3′	Target	t_melt_	Amplicon size (bp)^1^
**Screening PCR**	yjiD-yjiE FP	ttacttgtttcaggcatgcc	GimA locus	57	Variable
	yjiD-yjiE RP	atgaatcccgttgccgaacg			
**Long Range PCR** 2	yjiD-yjiE FP	ttacttgtttcaggcatgcc	Long range fragment 1	56	10.549
	gclKGimA3 RP	tgaatgagttacgccagtgtg			
	cniTGimA2 FP	ggacactgtactgtgggtta	Long range fragment 2	60	10.514
	yjiD-yjiE RP	atgaatcccgttgccgaacg			
***ibeA*** ** sequence analysis**	ibeAFr1F	gtattagcatgatgttgcttg	*ibeA* fragment 1	55	∼900
	ibeAFr1R	attttcttcatccgcccccc			
	ibeAFr2F	agctaatgtgtatgacaacc	*ibeA* Fragment 2	55	∼900
	ibeAFr2R	aaaaatgatcggtgtaagcg			
**Probe generation** 2	pptE FP	atgattgaggttcctgccgc	*pptE*	57	342
	pptE RP	ttagtgggaggttctgattgc			
	pdaKGimA1 FP	tcggtttgtttgcaccacac	*pdaK*/*cgrD*	57	571
	cgrDGimA2 RP	ccggtcacaaattcatctgca			
	cniTGimA2 FP	ggacactgtactgtgggtta	*cniT*/*gclK*	57	475
	gclKGimA3 RP	tgaatgagttacgccagtgtg			
	gclA FP	aatatagcgtgtctccggtcc	*gclA*	57	600
	gclA RP	aagcgagtgaattgttgcg			
	ibeA FP	tggaacccgctcgtaatatac	*ibeA*	57	1270
	ibeA RP	tacgccattttgctgtaagcg			

1 Based on strains RS218 (AF289032), APEC O1 (CP000468) and UTI89 (CP000243).

2 For a graphical overview of probe location see Figure 1.

### Analysis of the GimA locus: DNA-DNA hybridization analyses

DNA-DNA-hybridization analyses for the validation of PCR results were performed using probes for the GimA-related genes *gclA*, *pptE*, *pdaK*/*cgrD*, *cniT*/*gclK*, and *ibeA*. Oligonucleotide primers used for probe synthesis are listed in Table 1 and probe binding sites are illustrated in Figure 1. Hybridization was performed with digoxigenin (DIG)-dUTP-labeled probes using the PCR DIG probe synthesis kit and the Roche Labeling and Detection Kit (Roche Molecular Biochemicals, Mannheim, Germany) according to the manufacturer's instructions. DNA of NMEC strain RS218 used as a positive control in each reaction.

### Phylogenetic analysis

The sequences of the seven genes used for multi locus sequence typing (MLST) of the strains were aligned, trimmed to a uniform size and assembled in an extended multi-FASTA format (XMFA) in seven blocks, one for each gene. Phylogenetic relationships between the distinct sequence types were calculated using the previously described ClonalFrame v1.1 software (http://www2.warwick.ac.uk/fac/sci/statistics/staff/research/didelot/clonalframe/) [37]. The basis for this software is a model of genetic diversification that takes into account recombination processes that are expected to occur in bacterial populations such as *E. coli*. This allows deducing the phylogenetic relationships within bacterial populations based on the sequences of multiple MLST data sets, even considering that parts of them could underlie recombination effects.

ClonalFrame analysis was performed on the concatenated MLST sequences with the default parameters of 50000 MCMC iterations after 50000 burn-in iterations resulting in a majority-rule consensus tree which was then visualized using MEGA version 4 [38].

### STRUCTURE analysis

All sequence types (STs) included in the present study were assigned to one of the four major phylogenetic groups A, B1, B2 and D, as well as to recombinant groups AxB1 and ABD using the software Structure 2.2 (http://pritch.bsd.uchicago.edu/software/structure2_2.html), [39], [40] applying cut-off values described previously [25].

### Sequence analysis of *ibeA* and computational analyses

Thirty-four *E. coli* strains, representing the distribution of *ibeA* in the MLST background, were selected for the following investigation. Sequence analysis of the *ibeA* gene (1.341bp) was performed by sequencing two overlapping PCR fragments, including an up- and downstream *ibeA* region of about 100 bp. These fragments were generated with primer pairs ibeAFr1F/R and ibeAFr2F/R (Table 1). Except for an annealing temperature of 55°C and elongation time of 60 sec the amplification procedures were the same as described above. Sequencing was performed by AGOWA GmbH (Berlin, Germany) and sequence analysis was carried out using the assembler module of the Kodon software (Version 3.6 Applied Maths, Sint Martens-Latem, Belgium). Alignments and dendrograms were generated with the software using the Maximum Parsimony algorithm. Bootstrap values were computed with 1000 replicas.

### Evolutionary analysis of *ibeA* sequences derived from STC95 strains

Rates of non-synonymous (Dn) and synonymous (Ds) mutations were calculated using DnaSP 4.50.3 software available at http://www.ub.es/dnasp/ [41] in order to determine the Dn/Ds ratio for the *ibeA* locus and concatenated MLST sequence data sets of *E. coli* strains allocated to STC95 (n = 21). The results of the Dn/Ds ratio calculation were confirmed applying the single likelihood ancestor counting (SLAC) method. For SLAC analysis the web based analysis application available at http://www.datamonkey.org was used [42].

### Statistical Analysis

Significance of associations between pathotypes and GimA locus patterns was determined by a *χ* ^2^ test, using SPSS15 (SPSS Inc., Chicago, IL, USA).

## Results

### GimA locus analysis

Genetic characterization of the GimA locus by DNA-DNA hybridization and PCR analyses revealed variable sizes of the locus, schematically illustrated in Figure 1: a first pattern contains the complete GimA (∼20.3 kb) (GimA+) while a second pattern is characterized by the presence of a 342 bp remnant of *pptE* (GimA remnant). The latter one displays 100% identity to the terminal region of *pptE* of NMEC strain RS218 (*pptE* synonymous with *ptnI*) and APEC O1 strain, both of which harbour the complete *pptE* that is 2.382 bp in size and constitutes the first gene in the first operon of GimA (GimA1). The third variant of the locus does not contain any GimA related sequences and was therefore designated GimA-.

Analogous to the PCR approach, *in silico* analysis of the publicly available *E. coli* genomes identified the three patterns described above. *E. coli* strains RS218 (NMEC, ST95 of STC95), APEC O1 (APEC, ST95 of STC95), IMT5155 (APEC, ST140 of STC95) and UTI89 (UPEC, ST95 of STC95), display the GimA+ pattern, while UPEC strains CFT073 (ST73 of STC73), F11 (ST127, no complex), and 536 (ST127, no complex) possess a GimA remnant, while K-12 strain MG1655 (ST10 of STC10) is GimA-.

### Distribution of GimA variants among the *E. coli* population

**Figure 2:**
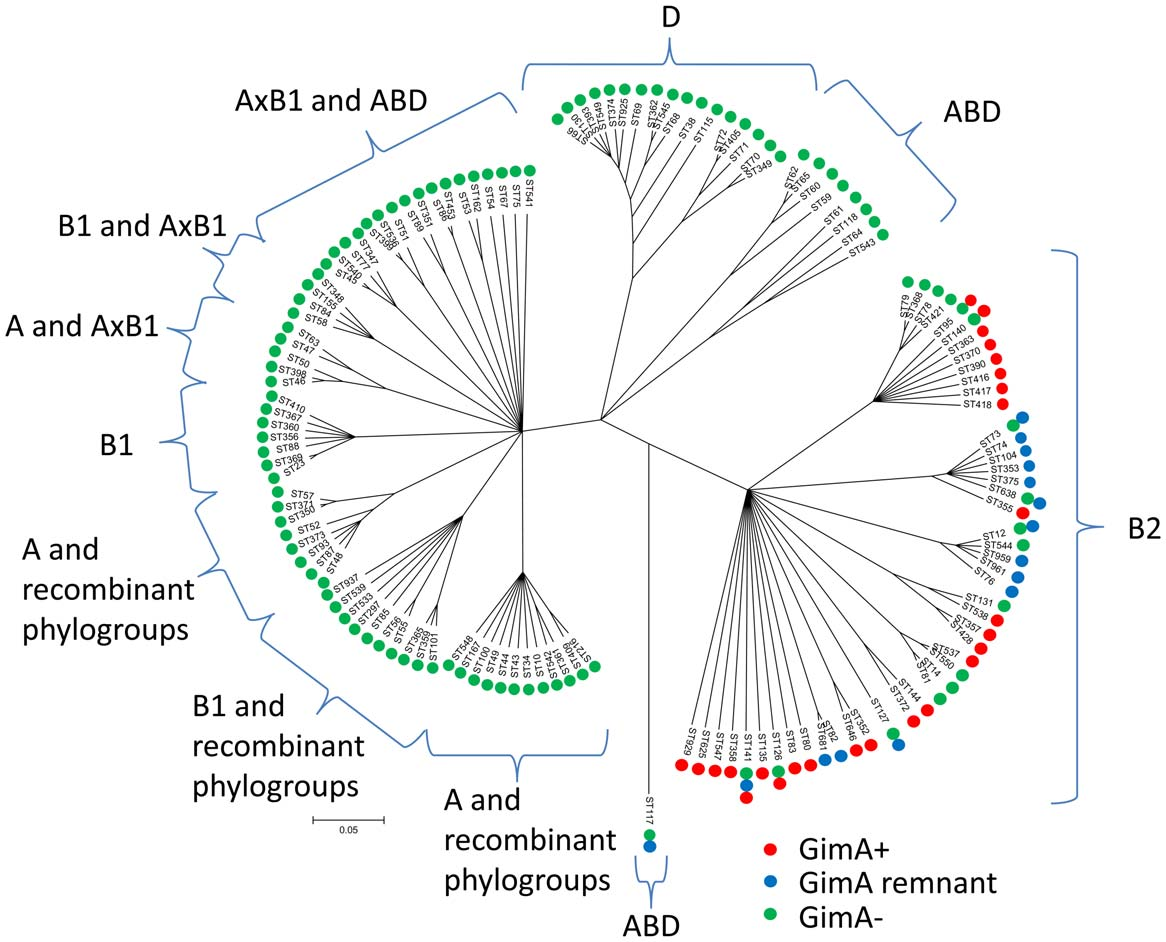
Majority-rule consensus tree of the concatenated MLST sequences as calculated by ClonalFrame depicted with MEGA4. The label presents ST and GimA locus patterns found within the ST (GimA+ red dots, GimA remnant blue dots and GimA- green dots). Phylogenetic groups (A, B1, B2, D, AxB1, and ABD) as determined by STRUCTURE analysis of the MLST sequence data sets are indicated by curly brackets.

Sixty-six (16.1%) of 410 strains tested were found to be GimA+, 80 strains (19.5%) possessed the GimA remnant, and 264 strains (64.4%) were GimA-. Figure 1 depicts the composition of the three patterns in more detail and Figure 2 displays the three patterns with regard to the phylogenetic background of respective strains. Among the 72 strains of the ECOR collection two strains (2.8%) were GimA+ and 62 (86.1%) GimA-, while eight strains (11.1%) harbored the GimA remnant. The results for all strains tested are given in Table S1.

The phylogenetic tree resulting from the ClonalFrame analysis was associated with phylogenetic groups as determined by STRUCTURE analysis. Each ST was assigned to one of the major phylogenetic groups (A, B1, B2 and D) or to a recombinant group (AxB1 and ABD) (Figure 2). Interestingly, GimA+ and GimA remnant strains occurred almost exclusively in ancestral group B2 with the only exception that GimA remnants were also present in 92.3% of a total of 13 strains belonging to ST117, which is affiliated to the recombinant phylogenetic group ABD [25]. The remaining phylogenetic groups (i.e. A, B1, and D) as well as recombinant groups AxB1 and ABD (except for ST117) were all GimA-.

While no significant associations between the presence of a certain GimA variant in a strain and the original host could be observed there was statistical support (p<0.05) for positive associations of GimA variants with different pathotypes (Table 2). Among group B2 APEC and NMEC strains high proportions of GimA+ strains (54.7% and 55.6%, respectively) were observed, whereas the GimA remnant was only present in low percentages (7.1% and 5.6%, respectively). Conversely, 51.0% of UPEC B2 strains harbored a GimA remnant, while only moderate proportions (22.4%) were GimA+. Commensal strains were predominantly correlated with the GimA- pattern, while only few strains (n = 10), albeit all affiliated to phylogenetic group B2, harbored a complete GimA. Detailed results of phylogenetic grouping and analysis of the GimA locus of ExPEC and commensal strains are given in Table S1.

**Table 2:** Distribution of the GimA locus patterns and pathotypes among 410 extraintestinal pathogenic and fecal *E. coli* strains.

	GimA locus pattern			
Pathotype	GimA+ (n) [B2/non B2]	GimA remnant (n)^2^ [B2/non B2]	GimA- (n) [B2/non B2]	Total No. of strains investigated [B2/non B2]
**APEC**	**23** (23/0)	13 (3/10)	62 (16/46)	98 (42/56)
**NMEC**	**10** (10/0)	1 (1/0)	14 (7/7)	25 (18/7)
**UPEC**	22 (22/0)	**52** (50/2)	66 (26/40)	140 (98/42)
**SEPEC**	1 (1/0)	5 (5/0)	22 (4/18)	28 (10/18)
**Human_fecal_**	9 (9/0)	6 (6/0)	**71** (16/55)	86 (31/55)
**Animal_fecal_**	1 (1/0)	3 (3/0)	**29** (0/29)	33 (4/29)
**Total No. of strains investigated**	66 (66/0)	80 (68/12)	264 (69/195)	410 (203/207)

Significant associations (chi square test, p<0.05)^1^ are highlighted by bold numbers.

1 Significant associations of the prevalence of a given GimA pattern in a given pathotype compared with all other pathotypes (p<0.05) are highlighted by bold numbers.

2 All 12 non-B2 strains belong to phylogenetic group ABD and sequence type (ST) 117.

### Sequence analysis of *ibeA*

**Figure 3:**
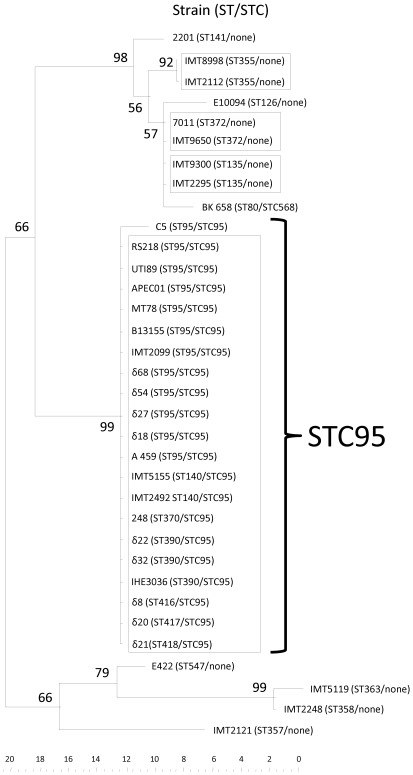
Maximum Parsimony tree constructed from *ibeA* sequence data (identical *ibeA* sequences are indicated by boxes).

The nucleotide sequence variation of the *ibeA* gene of 34 representative *E. coli* B2 strains allotted to 17 different STs were investigated. In a dendrogram based on these *ibeA* sequences, 12 distinct groups representing 12 allelic variants were identified (Figure 3). Except for NMEC strain C5 (ST95), all isolates belonging to ST95 (n = 11) harbored a 100% identical *ibeA* sequence. The *ibeA* sequence derived from NMEC strain C5 merely differed in two nucleotide positions from the other ST95 strains. Strains belonging to STC95 (in the present study ST95, ST140, ST370, ST390, ST416, ST 417, and ST418) also harbor identical *ibeA* sequences. Moreover, other STs that were represented by two isolates each (ST355, ST372, and ST135) also exhibited unique *ibeA* sequences. The presence of the same *ibeA* alleles in strains with identical STs (except for strain C5) might principally indicate a linked evolution of house keeping genes and *ibeA* and thus an absence of horizontal transfer of *ibeA*.

### Evolutionary analysis of *ibeA* sequences derived from STC95 strains (n = 21)

We determined the ratio of the non synonymous mutation rate (Dn) to the synonymous mutation rate (Ds). A Dn/Ds ratio ≤1 indicates neutral selection favoring amino acid conservation, while a Dn/Ds ratio >1 indicates positive selection, favoring amino acid substitutions [43], [44]. As STC95 has been shown to be an important ExPEC lineage [22], [27], [45], we chose this complex for a detailed analysis of the evolution of *ibeA* in its phylogenetic background. Due to its pathogenic role in systemic *E. coli* infections in chickens and meningitis in infants [12], [17] IbeA might be subjected to a selective pressure driven by host-protein interactions. Thus, a higher genetic diversity of *ibeA* compared to house keeping genes used for MLST analysis would not be unexpected. Within seven STC95-derived MLST sequences identified in the present the study we found an average of 1.43±0.58 synonymous mutations compared with 0.29±0.45 non synonymous mutations, resulting in Ds = 0.0017 and Dn = 0.0001, respectively. The Dn/Ds ratio of the concatenated MLST sequences yielded 0.0588, indicating neutral selection.

Two allelic variants have been observed among 21 *ibeA* sequences coming from STC95 strains. It is interesting to note the one allelic variant, which differed in two nucleotides from the other 20 sequences was exclusively present in NMEC strain C5. The differences were due to one synonymous (Ds = 0.0032) and one non synonymous mutation (Dn = 0.001) and resulted in a Dn/Ds ratio of 0.3125 once again indicating neutral selection. To confirm the occurrence of neutral selection on *ibeA* sequences derived from STC95 strains a SLAC analysis was carried out. No positively selected site was found and the mean Dn/Ds ratio corroborated the existence of neutral selection.

## Discussion

Translocation of the brain blood barrier is one of the most important steps in the establishment of neonatal meningitis caused by bacterial pathogens, including newborn meningitic *E. coli* [9]. In case of NMEC, a significant role in this process is attributed to a factor encoded by *ibeA*, which is located on a genomic island, termed GimA, constituted by four operons (GimA1–4) [11], [17]. Our study was initially performed to characterize GimA in more detail, with special emphasis on its genetic composition and its distribution among a collection of extraintestinal pathogenic and fecal *E. coli* strains. We observed three different patterns (GimA+, GimA remnant and GimA-) associated with the GimA locus, by that unraveling the polymorphic nature of this chromosomal region for the first time. The occurrence of genotypic correlates in certain regions of the *E. coli* chromosome [e.g. *mutS*-*rpoS* Region [46]] and PAIs [e.g. the LEE (locus of enterocyte effacement) locus [47], [48]] is well known. In case of the LEE this may be the result of a recurrent loss and acquisition of genes (patchwork model), or of a loss of parts of the locus by deletion events subsequent to its primary acquisition as an entity [49]. Such genetic variations have also been found to occur in other bacterial species [50] [51] and is a prominent feature of Staphylococcus aureus (SCC*mec*). Likewise, we have identified genetic variations in the putative insertion locus of GimA (between *yjiD*-*yjiE*) of ExPEC and fecal *E. coli* strains.

Genomic islands or pathogenicity islands (PAI) are chromosomal or episomal regions in pathogenic bacteria, associated with (i) virulence associated genes (VAGs),(ii) varying G+C contents compared with the host chromosome, (iii) flanking mobility and insertion elements, such as IS elements, integrases, transposases, direct repeats, and (iv) tRNA genes [52], [53], [54].

Some characteristics like the absence of direct repeats surrounding GimA, high frequency in pathogenic *E. coli* strains, and a reduced G+C content (46.2%) speak for the fact that GimA is a PAI; however, initial *in silico* analysis of the GimA genomic region of UPEC strain UTI89 and APEC O1 strain has revealed the absence of mobility and insertion elements 10 kb up- and downstream of GimA, which contradicts this assumption. We also found no association with tRNA genes, with the nearest tRNA genes being *leuX* (141 kb upstream in UTI89 and 22 kb in APEC O1) and *leuQ* (44 kb downstream in both strains).

We could demonstrate that the three GimA variants were always located between genes *yjiD* and *yjiE*, indicating a vertical spread of GimA in accordance with the phylogenetic background rather than a horizontal transfer. Genomic islands transferred horizontally are usually characterized by the occurrence of different allelic variants of genes located on these islands within a certain phylogenetic complex [55], [56]. Such variants, derived by multiple insertion events, might subsequently diversify by further point mutations as a kind of pathoadaptation, as has been reported for adhesin genes *fimH* and *papC* [56]. We observed only one *ibeA* allele among STC95 strains, which may perhaps indicate an analogy between *ibeA* alleles and certain phylogenetic lineages (Figure 3). However, a single NMEC strain C5 of the STC95 showed an additional allele through the presence of two SNPs. Of note, that these two SNPs occurred in the same strain, suggests a potential horizontal transfer event, rather than a double point mutation, even though this remains unclear. It may therefore contradict the theory of a lack of horizontal gene transfer; however, the overall finding that no pathoadaptive mutations occur in the *ibeA* gene still holds true given the number of strains with an identical allele.

A possible concern could be that the small size of *ibeA*, together with the short evolutionary time frame that is supposed to have influenced strains within STC95, may limit the chance to detect any selection signal, whether positive or neutral; however, previous studies have shown the presence of a positive selection in adhesin genes of a similar size [56], [57].

One could assume that at a certain time point GimA was stably integrated into the *E. coli* chromosome, with deletion events rather than insertion events being the likely way that these three patterns could have emerged. This may be the reason for the exclusive association of GimA with B2 strains in this study, indicating an early integration of GimA with regard to *E. coli* phylogeny, thereby suggesting the presence of GimA as an ancestral trait within this group. The absence or cryptic character of mobility elements in the surrounding region of GimA further strengthens this suggestion.

The GimA remnant was also found to be almost exclusively associated with the B2 phylogenetic group, with the exception of a group of strains belonging to ST117. It has been previously reported that apart from the B2 group, there exists a subgroup within the phylogenetic group D which also belongs to the so called basal group [58]. Further analysis assigned ST117 to recombinant phylogenetic group ABD. However, the PCR based method for rapid phylogenetic typing affiliates ST117 strains to phylogenetic group D [59], thus possibly resulting in the basal subgroup being represented by strains of ST117. Another possibility would be that the GimA remnant might have been acquired by recombination from a B2 parent strain, but this still needs to be clarified.

Dobrindt et al. reported that “en bloc” gene acquisition and subsequent gene loss can be regarded as a mechanism of genome optimization reflecting the lifestyle of a microorganism [60]. Considerable genome reduction has been reported for various bacterial species, including bacterial endosymbionts, phytopathogens and zoopathogens [61], [62], [63], [64]. The maintenance of the GimA remnant concomitant with the rest of GimA undergoing a deletion process might fit into what has been proposed as a ‘change-of-function’ mechanism in bacterial pathogens [65]. The idea behind this is that not only the acquisition of additional genes coding for specific virulence factors (‘gain-of-function’ mechanism) but also a functional modification or loss of pre-existing genetic material could direct bacterial evolution toward a more pathogenic phenotype, often driven by strong selective pressure upon the bacterial clone in the respective virulence niche, as for example the urinary tract [65].

The distribution of GimA variants between phylogenetic groups might lead us to believe that GimA remnants and GimA negative strains could have arisen through two subsequent deletion events (Figure 1), although the reasons behind this are still unclear.

On the one hand, we found strains belonging to the pathotypes NMEC and APEC significantly more likely to harbour pattern GimA+. This points towards the importance of GimA among these pathotypes, as has been published previously for a number of GimA-associated genes [12], [66], [67], [68], [69]. On the other hand UPEC strains were highly associated with the GimA remnant. *In silico* analysis of the publicly available sequence data of UPEC strains CFT073, 536 and F11 also revealed the presence of a GimA remnant in these strains. A subsequent open reading frame analysis using the Kodon software identified the 342 bp remnant as an *orf* including both a start and a stop codon. While the stop codon is identical to that of the regular *pptE*, the ATG at position 2.340 coding for Met in the parent *pptE* serves as the start codon in the remnant. Additionally, using the BPROM promoter prediction tool (provided on http://linux1.softberry.com) we could identify a transcriptional factor binding site upstream of the start codon (data not shown). However, it is not known whether this fragment is expressed *in vivo* nor under which conditions this would occur.

Our genotyping data suggest a correlation of GimA variants with a habitat-specific pathogenicity, although experimental evidence remains to be provided. Functional assays are clearly needed to determine whether such ‘change-of-function’ mechanisms have led to the reductive evolution in the GimA locus, with particular emphasis on unraveling the pathogenic role of the GimA remnant in uropathogenicity.

### Concluding remarks

We have identified three different patterns of the GimA locus (GimA+, GimA remnant and GimA-) associated with a core-genome analogous evolution. To our knowledge this is the first report about the existence of the GimA remnant. Many findings allow us to conclude that GimA should henceforth not be classified as a genomic island but rather given a modified term, namely GimA locus.

## Supporting Information

Table S1 *E. coli* strains used in this study: Sequence type (ST) and ST complexes, GimA locus pattern (GimA+, GimA remnant and GimA-) and EcoR phylogroup based on STRUCTURE analysis using MLST sequence data.
(0.66 MB DOC)
