# Supplementary material for: The GimA Locus of Extraintestinal Pathogenic E. coli: Does Reductive Evolution Correlate with Habitat and Pathotype?
Source: PLoS One. 2010 May 28;5(5):e10877. doi: 10.1371/journal.pone.0010877 (PMC2878320; doi:10.1371/journal.pone.0010877)
Supplement: Table S1 — E. coli strains used in this study: Sequence type (ST) and ST complexes, GimA locus pattern (GimA+, GimA remnant and GimA-) and EcoR phylogroup based on STRUCTURE analysis using MLST sequence data. (0.66 MB DOC) [file pone.0010877.s001.doc]

| **Pathovar** | **Designation** | **Sequence type** | **Sequence type complex** | **ECOR group** | **GimA locus pattern** | **Host** |
| --- | --- | --- | --- | --- | --- | --- |
| APEC | IMT2099 | 95 | 95 | B2 | GimA+ | animal |
| APEC | IMT2112 | 355 | None | B2 | GimA+ | animal |
| APEC | IMT2121 | 357 | None | B2 | GimA+ | animal |
| APEC | IMT2248 | 358 | None | B2 | GimA+ | animal |
| APEC | IMT2288 | 140 | 95 | B2 | GimA+ | animal |
| APEC | IMT2295 | 135 | None | B2 | GimA+ | animal |
| APEC | IMT2297 | 95 | 95 | B2 | GimA+ | animal |
| APEC | IMT2469 | 140 | 95 | B2 | GimA+ | animal |
| APEC | IMT2470 | 95 | 95 | B2 | GimA+ | animal |
| APEC | IMT2492 | 140 | 95 | B2 | GimA+ | animal |
| APEC | IMT2545 | 95 | 95 | B2 | GimA+ | animal |
| APEC | IMT4514 | 352 | None | B2 | GimA+ | animal |
| APEC | IMT4534 | 140 | 95 | B2 | GimA+ | animal |
| APEC | IMT5119 | 363 | None | B2 | GimA+ | animal |
| APEC | IMT5155 | 140 | 95 | B2 | GimA+ | animal |
| APEC | 2201 | 141 | None | B2 | GimA+ | animal |
| APEC | IMT8994 | 355 | None | B2 | GimA+ | animal |
| APEC | IMT8998 | 355 | None | B2 | GimA+ | animal |
| NMEC | IHE 3036 | 390 | 95 | B2 | GimA+ | human |
| NMEC | IHE 3080 | 390 | 95 | B2 | GimA+ | human |
| NMEC | BK658 | 80 | 568 | B2 | GimA+ | human |
| NMEC | B13155 | 95 | 95 | B2 | GimA+ | human |
| APEC | MT78 | 95 | 95 | B2 | GimA+ | animal |
| APEC | 248 | 370 | 95 | B2 | GimA+ | animal |
| APEC | 2363 | 135 | None | B2 | GimA+ | animal |
| UPEC | IMT9650 | 372 | None | B2 | GimA+ | animal |
| APEC | IMT9698 | 95 | 95 | B2 | GimA+ | animal |
| APEC | IMT9713 | 372 | None | B2 | GimA+ | animal |
| Human_fecal_ | δ8 | 416 | 95 | B2 | GimA+ | human |
| Human_fecal_ | δ18 | 95 | 95 | B2 | GimA+ | human |
| UPEC | δ20 | 417 | 95 | B2 | GimA+ | human |
| UPEC | δ21 | 418 | 95 | B2 | GimA+ | human |
| UPEC | δ22 | 390 | 95 | B2 | GimA+ | human |
| SePEC | δ27 | 95 | 95 | B2 | GimA+ | human |
| UPEC | δ32 | 390 | 95 | B2 | GimA+ | human |
| UPEC | δ54 | 95 | 95 | B2 | GimA+ | human |
| UPEC | δ68 | 95 | 95 | B2 | GimA+ | human |
| Human_fecal_ | E7370 | 538 | 538 | B2 | GimA+ | human |
| Human_fecal_ | E452 | 95 | 95 | B2 | GimA+ | human |
| Human_fecal_ | E10094 | 126 | None | B2 | GimA+ | human |
| Human_fecal_ | E475 | 537 | 14 | B2 | GimA+ | human |
| Human_fecal_ | E422 | 547 | None | B2 | GimA+ | human |
| Human_fecal_ | E478 | 428 | None | B2 | GimA+ | human |
| NMEC | RS218 | 95 | 95 | B2 | GimA+ | human |
| NMEC | A459 | 95 | 95 | B2 | GimA+ | human |
| UPEC | 15016 | 141 | None | B2 | GimA+ | animal |
| NMEC | C5 | 95 | 95 | B2 | GimA+ | human |
| NMEC | RS167 | 144 | None | B2 | GimA+ | human |
| NMEC | E334 | 144 | None | B2 | GimA+ | human |
| UPEC | IMT15029 | 372 | None | B2 | GimA+ | animal |
| UPEC | IMT15030 | 372 | None | B2 | GimA+ | animal |
| UPEC | IMT14984 | 372 | None | B2 | GimA+ | animal |
| UPEC | IMT14986 | 141 | None | B2 | GimA+ | animal |
| UPEC | IMT14989 | 141 | None | B2 | GimA+ | animal |
| UPEC | IMT14990 | 625 | None | B2 | GimA+ | animal |
| UPEC | IMT14994 | 372 | None | B2 | GimA+ | animal |
| UPEC | IMT14963 | 646 | None | B2 | GimA+ | animal |
| UPEC | IMT14964 | 646 | None | B2 | GimA+ | animal |
| UPEC | IMT14997 | 372 | None | B2 | GimA+ | animal |
| UPEC | IMT15001 | 141 | None | B2 | GimA+ | animal |
| NMEC | IHE3034 | 95 | 95 | B2 | GimA+ | human |
| UPEC | IMT15009 | 80 | 568 | B2 | GimA+ | animal |
| UPEC | IMT14954 | 141 | None | B2 | GimA+ | animal |
| UPEC | IMT14988 | 929 | None | B2 | GimA+ | animal |
| Human_fecal_ | ECOR-63 | 80 | 568 | B2 | GimA+ | human |
| Animal_fecal_ | ECOR-66 | 83 | none | B2 | GimA+ | animal |
| UPEC | IMT1200 | 353 | 73 | B2 | GimA remnant | human |
| APEC | IMT2102 | 117 | None | ABD | GimA remnant | animal |
| APEC | IMT2106 | 117 | None | ABD | GimA remnant | animal |
| APEC | IMT2477 | 141 | None | B2 | GimA remnant | animal |
| APEC | IMT2489 | 117 | None | ABD | GimA remnant | animal |
| APEC | IMT2490 | 117 | None | ABD | GimA remnant | animal |
| APEC | IMT2518 | 104 | 73 | B2 | GimA remnant | animal |
| APEC | IMT4516 | 117 | None | ABD | GimA remnant | animal |
| APEC | IMT4529 | 117 | None | ABD | GimA remnant | animal |
| APEC | IMT5144 | 141 | None | B2 | GimA remnant | animal |
| UPEC | 536 | 92 | None | B2 | GimA remnant | human |
| UPEC | AD110 | 73 | 73 | B2 | GimA remnant | human |
| UPEC | RZ422 | 127 | None | B2 | GimA remnant | human |
| UPEC | RZ479 | 127 | None | B2 | GimA remnant | human |
| UPEC | U-7 | 117 | None | ABD | GimA remnant | human |
| UPEC | C89 | 12 | 12 | B2 | GimA remnant | human |
| UPEC | CFTO73 | 73 | 73 | B2 | GimA remnant | human |
| UPEC | B 6985-2 | 73 | 73 | B2 | GimA remnant | human |
| UPEC | B 6993 | 141 | None | B2 | GimA remnant | human |
| UPEC | IMT9096 | 73 | 73 | B2 | GimA remnant | animal |
| UPEC | IMT9206 | 375 | 73 | B2 | GimA remnant | animal |
| APEC | IMT9242 | 117 | None | ABD | GimA remnant | animal |
| NMEC | E247 | 127 | None | B2 | GimA remnant | human |
| UPEC | IMT9289 | 73 | 73 | B2 | GimA remnant | human |
| APEC | V-Z | 117 | None | ABD | GimA remnant | animal |
| UPEC | IMT9402 | 104 | 73 | B2 | GimA remnant | animal |
| APEC | IMT9582 | 117 | None | ABD | GimA remnant | animal |
| APEC | IMT9586 | 117 | None | ABD | GimA remnant | animal |
| Human_fecal_ | E176 | 73 | 73 | B2 | GimA remnant | human |
| SEPEC | i484 | 73 | 73 | B2 | GimA remnant | human |
| SEPEC | F1 | 73 | 73 | B2 | GimA remnant | human |
| SEPEC | F385 | 73 | 73 | B2 | GimA remnant | human |
| SEPEC | F911 | 12 | 12 | B2 | GimA remnant | human |
| SEPEC | St5119 | 141 | None | B2 | GimA remnant | human |
| UPEC | U2388 | 73 | 73 | B2 | GimA remnant | human |
| UPEC | U2873 | 127 | None | B2 | GimA remnant | human |
| UPEC | U3145 | 73 | 73 | B2 | GimA remnant | human |
| UPEC | U3362 | 73 | 73 | B2 | GimA remnant | human |
| Human_fecal_ | E10091 | 141 | None | B2 | GimA remnant | human |
| Human_fecal_ | E471 | 73 | 73 | B2 | GimA remnant | human |
| UPEC | IMT14965 | 73 | 73 | B2 | GimA remnant | animal |
| UPEC | IMT14966 | 73 | 73 | B2 | GimA remnant | animal |
| UPEC | IMT14967 | 73 | 73 | B2 | GimA remnant | animal |
| UPEC | IMT14968 | 73 | 73 | B2 | GimA remnant | animal |
| UPEC | IMT14969 | 73 | 73 | B2 | GimA remnant | animal |
| UPEC | IMT15014 | 117 | None | ABD | GimA remnant | animal |
| UPEC | IMT15015 | 12 | 12 | B2 | GimA remnant | animal |
| UPEC | IMT14970 | 104 | 73 | B2 | GimA remnant | animal |
| UPEC | IMT15019 | 127 | None | B2 | GimA remnant | animal |
| UPEC | IMT15024 | 141 | None | B2 | GimA remnant | animal |
| UPEC | IMT15025 | 12 | 12 | B2 | GimA remnant | animal |
| UPEC | IMT15031 | 12 | 12 | B2 | GimA remnant | animal |
| UPEC | IMT15033 | 73 | 73 | B2 | GimA remnant | animal |
| UPEC | IMT14973 | 12 | 12 | B2 | GimA remnant | animal |
| UPEC | IMT14974 | 12 | 12 | B2 | GimA remnant | animal |
| UPEC | IMT14975 | 127 | None | B2 | GimA remnant | animal |
| UPEC | IMT14976 | 73 | 73 | B2 | GimA remnant | animal |
| UPEC | IMT14977 | 12 | 12 | B2 | GimA remnant | animal |
| UPEC | IMT14978 | 127 | None | B2 | GimA remnant | animal |
| UPEC | IMT14981 | 638 | 73 | B2 | GimA remnant | animal |
| UPEC | IMT14958 | 73 | 73 | B2 | GimA remnant | animal |
| UPEC | IMT14959 | 12 | 12 | B2 | GimA remnant | animal |
| UPEC | IMT14993 | 127 | None | B2 | GimA remnant | animal |
| UPEC | IMT14995 | 73 | 73 | B2 | GimA remnant | animal |
| UPEC | IMT14998 | 73 | 73 | B2 | GimA remnant | animal |
| UPEC | IMT14999 | 681 | None | B2 | GimA remnant | animal |
| UPEC | IMT15003 | 12 | 12 | B2 | GimA remnant | animal |
| UPEC | IMT15004 | 127 | None | B2 | GimA remnant | animal |
| UPEC | IMT15008 | 73 | 73 | B2 | GimA remnant | animal |
| UPEC | IMT15010 | 12 | 12 | B2 | GimA remnant | animal |
| UPEC | IMT14982 | 959 | None | B2 | GimA remnant | animal |
| UPEC | IMT15005 | 961 | None | B2 | GimA remnant | animal |
| Human_fecal_ | ECOR-51 | 73 | 73 | B2 | GimA remnant | human |
| Animal_fecal_ | ECOR-52 | 73 | 73 | B2 | GimA remnant | animal |
| Human_fecal_ | ECOR-54 | 73 | 73 | B2 | GimA remnant | human |
| UPEC | ECOR-55 | 74 | 73 | B2 | GimA remnant | human |
| Animal_fecal_ | ECOR-57 | 73 | 73 | B2 | GimA remnant | animal |
| Human_fecal_ | ECOR-59 | 76 | None | B2 | GimA remnant | human |
| UPEC | ECOR-60 | 12 | 12 | B2 | GimA remnant | human |
| Animal_fecal_ | ECOR-65 | 82 | None | B2 | GimA remnant | animal |
| APEC | IMT664 | 88 | 23 | B1 | GimA- | animal |
| APEC | IMT1431 | 88 | 23 | B1 | GimA- | animal |
| APEC | IMT1432 | 88 | 23 | B1 | GimA- | animal |
| APEC | IMT1433 | 88 | 23 | B1 | GimA- | animal |
| APEC | IMT1930 | 88 | 23 | B1 | GimA- | animal |
| APEC | IMT1932 | 23 | 23 | B1 | GimA- | animal |
| APEC | IMT1935 | 23 | 23 | B1 | GimA- | animal |
| APEC | IMT1936 | 23 | 23 | B1 | GimA- | animal |
| APEC | IMT1937 | 155 | 155 | AxB1 | GimA- | animal |
| APEC | IMT1938 | 155 | 155 | AxB1 | GimA- | animal |
| APEC | IMT1939 | 155 | 155 | AxB1 | GimA- | animal |
| APEC | IMT2087 | 95 | 95 | B2 | GimA- | animal |
| APEC | IMT2089 | 23 | 23 | B1 | GimA- | animal |
| APEC | IMT2092 | 347 | None | AxB1 | GimA- | animal |
| APEC | IMT2094 | 23 | 23 | B1 | GimA- | animal |
| APEC | IMT2095 | 162 | 469 | AxB1 | GimA- | animal |
| APEC | IMT2097 | 95 | 95 | B2 | GimA- | animal |
| APEC | IMT2098 | 115 | None | D | GimA- | animal |
| APEC | IMT2101 | 23 | 23 | B1 | GimA- | animal |
| APEC | IMT2104 | 348 | 156 | B1 | GimA- | animal |
| APEC | IMT2105 | 117 | None | ABD | GimA- | animal |
| APEC | IMT2108 | 95 | 95 | B2 | GimA- | animal |
| APEC | IMT2111 | 38 | 38 | D | GimA- | animal |
| APEC | IMT2113 | 101 | 101 | B1 | GimA- | animal |
| APEC | IMT2120 | 356 | 23 | B1 | GimA- | animal |
| APEC | IMT2125 | 23 | 23 | B1 | GimA- | animal |
| APEC | IMT2250 | 95 | 95 | B2 | GimA- | animal |
| APEC | IMT2251 | 95 | 95 | B2 | GimA- | animal |
| APEC | IMT2254 | 126 | None | B2 | GimA- | animal |
| APEC | IMT2261 | 349 | 349 | D | GimA- | animal |
| APEC | IMT2263 | 93 | 168 | AxB1 | GimA- | animal |
| APEC | IMT2264 | 350 | 350 | ABD | GimA- | animal |
| APEC | IMT2265 | 351 | None | ABD | GimA- | animal |
| APEC | IMT2278 | 140 | 95 | B2 | GimA- | animal |
| APEC | IMT2283 | 23 | 23 | B1 | GimA- | animal |
| APEC | IMT2294 | 115 | None | D | GimA- | animal |
| APEC | IMT2352 | 369 | 23 | B1 | GimA- | animal |
| APEC | IMT2467 | 23 | 23 | B1 | GimA- | animal |
| APEC | IMT2491 | 95 | 95 | B2 | GimA- | animal |
| APEC | IMT2532 | 95 | 95 | B2 | GimA- | animal |
| APEC | IMT4517 | 95 | 95 | B2 | GimA- | animal |
| APEC | IMT4518 | 95 | 95 | B2 | GimA- | animal |
| APEC | IMT4525 | 95 | 95 | B2 | GimA- | animal |
| APEC | IMT4537 | 118 | None | ABD | GimA- | animal |
| APEC | IMT4541 | 88 | 23 | B1 | GimA- | animal |
| APEC | IMT5110 | 100 | 165 | A | GimA- | animal |
| APEC | IMT5132 | 365 | 101 | AxB1 | GimA- | animal |
| APEC | IMT5215 | 93 | 168 | AxB1 | GimA- | animal |
| APEC | IMT5494 | 359 | None | AxB1 | GimA- | animal |
| UPEC | IMT6008 | 367 | 23 | B1 | GimA- | animal |
| UPEC | J96 | 12 | 12 | B2 | GimA- | human |
| UPEC | 764 | 14 | 14 | B2 | GimA- | human |
| UPEC | C97 | 10 | 10 | A | GimA- | human |
| UPEC | C90 | 70 | None | D | GimA- | human |
| UPEC | C70 | 14 | 14 | B2 | GimA- | human |
| UPEC | ABV84 | 360 | 23 | B1 | GimA- | human |
| APEC | 2164 | 368 | 95 | B2 | GimA- | animal |
| APEC | 2446 | 95 | 95 | B2 | GimA- | animal |
| APEC | IMT9003 | 369 | 23 | B1 | GimA- | animal |
| UPEC | B 6977-1 | 131 | None | B2 | GimA- | human |
| UPEC | B 6998 | 361 | None | AxB1 | GimA- | human |
| UPEC | B 7001 | 362 | None | D | GimA- | human |
| UPEC | IMT9179 | 374 | 31 | D | GimA- | animal |
| UPEC | IMT9227 | 68 | None | D | GimA- | animal |
| APEC | IMT9232 | 95 | 95 | B2 | GimA- | animal |
| APEC | IMT9238 | 10 | 10 | A | GimA- | animal |
| APEC | IMT9241 | 95 | 95 | B2 | GimA- | animal |
| APEC | IMT9250 | 57 | 350 | ABD | GimA- | animal |
| NMEC | B10363 | 95 | 95 | B2 | GimA- | human |
| NMEC | VE239 | 62 | None | ABD | GimA- | human |
| NMEC | VE1140 | 95 | 95 | B2 | GimA- | human |
| NMEC | E817 | 127 | None | B2 | GimA- | human |
| UPEC | IMT9280 | 95 | 95 | B2 | GimA- | human |
| APEC | IMT9581 | 23 | 23 | B1 | GimA- | animal |
| APEC | IMT9583 | 23 | 23 | B1 | GimA- | animal |
| APEC | IMT9584 | 23 | 23 | B1 | GimA- | animal |
| APEC | IMT9588 | 371 | 350 | ABD | GimA- | animal |
| APEC | IMT9706 | 10 | 10 | A | GimA- | animal |
| APEC | IMT9712 | 373 | 168 | A | GimA- | animal |
| Animal_fecal_ | IMT10666 | 58 | 155 | AxB1 | GimA- | animal |
| Human_fecal_ | δ1 | 95 | 95 | B2 | GimA- | human |
| Human_fecal_ | δ9 | 95 | 95 | B2 | GimA- | human |
| UPEC | δ11 | 95 | 95 | B2 | GimA- | human |
| UPEC | δ15 | 421 | 95 | B2 | GimA- | human |
| SEPEC | δ29 | 95 | 95 | B2 | GimA- | human |
| UPEC | δ30 | 95 | 95 | B2 | GimA- | human |
| Human_fecal_ | δ34 | 95 | 95 | B2 | GimA- | human |
| UPEC | δ37 | 95 | 95 | B2 | GimA- | human |
| SEPEC | δ39 | 95 | 95 | B2 | GimA- | human |
| Human_fecal_ | δ58 | 95 | 95 | B2 | GimA- | human |
| Human_fecal_ | δ62 | 95 | 95 | B2 | GimA- | human |
| Human_fecal_ | δ64 | 95 | 95 | B2 | GimA- | human |
| UPEC | δ67 | 95 | 95 | B2 | GimA- | human |
| Human_fecal_ | E10097 | 10 | 10 | A | GimA- | human |
| Human_fecal_ | E10077 | 162 | 469 | AxB1 | GimA- | human |
| Human_fecal_ | E10096 | 167 | 10 | A | GimA- | human |
| Human_fecal_ | E10099 | 34 | 10 | A | GimA- | human |
| Human_fecal_ | E10100 | 405 | 405 | D | GimA- | human |
| Human_fecal_ | E10084 | 38 | 38 | D | GimA- | human |
| Human_fecal_ | E10082 | 10 | 10 | A | GimA- | human |
| Human_fecal_ | E10095 | 549 | None | D | GimA- | human |
| Human_fecal_ | E10085 | 10 | 10 | A | GimA- | human |
| Human_fecal_ | E10098 | 69 | 69 | D | GimA- | human |
| Human_fecal_ | E476 | 10 | 10 | A | GimA- | human |
| Human_fecal_ | E164 | 10 | 10 | A | GimA- | human |
| Human_fecal_ | E167 | 10 | 10 | A | GimA- | human |
| Human_fecal_ | E291 | 10 | 10 | A | GimA- | human |
| Human_fecal_ | E264 | 399 | 399 | AxB1 | GimA- | human |
| Human_fecal_ | E424 | 38 | 38 | D | GimA- | human |
| Human_fecal_ | E179 | 350 | 350 | ABD | GimA- | human |
| Human_fecal_ | E175 | 95 | 95 | B2 | GimA- | human |
| SEPEC | F287 | 10 | 10 | A | GimA- | human |
| SEPEC | F557 | 23 | 23 | B1 | GimA- | human |
| SEPEC | F560 | 544 | 12 | B2 | GimA- | human |
| SEPEC | F569 | 88 | 23 | B1 | GimA- | human |
| SEPEC | F645 | 62 | None | ABD | GimA- | human |
| SEPEC | F742 | 539 | None | B1 | GimA- | human |
| SEPEC | F775 | 540 | None | AxB1 | GimA- | human |
| SEPEC | F785 | 10 | 10 | A | GimA- | human |
| SEPEC | F905 | 10 | 10 | A | GimA- | human |
| SEPEC | F1251 | 10 | 10 | A | GimA- | human |
| SEPEC | St4723 | 297 | None | B1 | GimA- | human |
| SEPEC | St5679 | 38 | 38 | D | GimA- | human |
| SEPEC | V9261 | 88 | 23 | B1 | GimA- | human |
| SEPEC | V9343 | 216 | None | A | GimA- | human |
| SEPEC | V10744 | 88 | 23 | B1 | GimA- | human |
| SEPEC | W7483 | 73 | 73 | B2 | GimA- | human |
| SEPEC | W7716 | 545 | None | D | GimA- | human |
| SEPEC | W8987 | 542 | None | ABD | GimA- | human |
| SEPEC | W9763 | 46 | 46 | AxB1 | GimA- | human |
| SEPEC | W9887 | 48 | 10 | A | GimA- | human |
| UPEC | U2183 | 453 | 86 | ABD | GimA- | human |
| UPEC | U2366 | 10 | 10 | A | GimA- | human |
| UPEC | U3104 | 533 | None | B1 | GimA- | human |
| UPEC | U3372 | 409 | None | AxB1 | GimA- | human |
| UPEC | U3407 | 95 | 95 | B2 | GimA- | human |
| UPEC | U3292 | 130 | 31 | D | GimA- | human |
| UPEC | U3454 | 95 | 95 | B2 | GimA- | human |
| UPEC | U3633 | 10 | 10 | A | GimA- | human |
| UPEC | U3622 | 88 | 23 | B1 | GimA- | human |
| UPEC | U4191 | 93 | 168 | AxB1 | GimA- | human |
| UPEC | U4252 | 48 | 10 | A | GimA- | human |
| UPEC | U4418 | 10 | 10 | A | GimA- | human |
| UPEC | U4437 | 127 | None | B2 | GimA- | human |
| UPEC | U4417 | 398 | 398 | AxB1 | GimA- | human |
| UPEC | U4409 | 393 | 31 | D | GimA- | human |
| UPEC | U5107 | 10 | 10 | A | GimA- | human |
| UPEC | U5033 | 93 | 168 | AxB1 | GimA- | human |
| UPEC | U5070 | 69 | 69 | D | GimA- | human |
| Human_fecal_ | E10093 | 405 | 405 | D | GimA- | human |
| Human_fecal_ | E10089 | 543 | None | ABD | GimA- | human |
| Human_fecal_ | E10092 | 536 | 399 | ABD | GimA- | human |
| Human_fecal_ | E10086 | 541 | 522 | A | GimA- | human |
| Human_fecal_ | E10090 | 10 | 10 | A | GimA- | human |
| Human_fecal_ | E472 | 68 | None | D | GimA- | human |
| Human_fecal_ | E460 | 38 | 38 | D | GimA- | human |
| Human_fecal_ | E165 | 95 | 95 | B2 | GimA- | human |
| Human_fecal_ | E166 | 10 | 10 | A | GimA- | human |
| Human_fecal_ | E292 | 10 | 10 | A | GimA- | human |
| Human_fecal_ | E345 | 10 | 10 | A | GimA- | human |
| Human_fecal_ | E182 | 73 | 73 | B2 | GimA- | human |
| Human_fecal_ | E444 | 548 | 10 | A | GimA- | human |
| Human_fecal_ | E457 | 95 | 95 | B2 | GimA- | human |
| Human_fecal_ | E177 | 95 | 95 | B2 | GimA- | human |
| Human_fecal_ | E178 | 95 | 95 | B2 | GimA- | human |
| Human_fecal_ | E180 | 10 | 10 | A | GimA- | human |
| Human_fecal_ | E173 | 393 | 31 | D | GimA- | human |
| Human_fecal_ | E174 | 348 | 156 | B1 | GimA- | human |
| Human_fecal_ | E464 | 550 | 14 | B2 | GimA- | human |
| Human_fecal_ | E467 | 88 | 23 | B1 | GimA- | human |
| NMEC | RS176 | 62 | None | ABD | GimA- | human |
| NMEC | C183-77 | 62 | None | ABD | GimA- | human |
| NMEC | IHE3055 | 95 | 95 | B2 | GimA- | human |
| NMEC | RS179 | 62 | None | ABD | GimA- | human |
| NMEC | EC10 | 62 | None | ABD | GimA- | human |
| NMEC | A90 | 95 | 95 | B2 | GimA- | human |
| NMEC | RS168 | 59 | 59 | ABD | GimA- | human |
| NMEC | S88 | 95 | 95 | B2 | GimA- | human |
| NMEC | S95 | 95 | 95 | B2 | GimA- | human |
| NMEC | E253 | 10 | 10 | A | GimA- | human |
| UPEC | IMT15017 | 10 | 10 | A | GimA- | animal |
| UPEC | IMT15021 | 73 | 73 | B2 | GimA- | animal |
| UPEC | IMT15022 | 115 | None | D | GimA- | animal |
| UPEC | IMT15028 | 12 | 12 | B2 | GimA- | animal |
| UPEC | IMT14972 | 10 | 10 | A | GimA- | animal |
| UPEC | IMT14955 | 12 | 12 | B2 | GimA- | animal |
| UPEC | IMT14979 | 73 | 73 | B2 | GimA- | animal |
| UPEC | IMT14980 | 638 | 73 | B2 | GimA- | animal |
| UPEC | IMT14983 | 539 | None | B1 | GimA- | animal |
| UPEC | IMT14985 | 12 | 12 | B2 | GimA- | animal |
| UPEC | IMT14987 | 75 | None | AxB1 | GimA- | animal |
| UPEC | IMT14991 | 12 | 12 | B2 | GimA- | animal |
| UPEC | IMT14961 | 12 | 12 | B2 | GimA- | animal |
| UPEC | IMT15000 | 95 | 95 | B2 | GimA- | animal |
| UPEC | IMT15002 | 410 | 23 | B1 | GimA- | animal |
| UPEC | IMT15006 | 88 | 23 | B1 | GimA- | animal |
| UPEC | IMT15007 | 141 | None | B2 | GimA- | animal |
| UPEC | IMT15011 | 10 | 10 | A | GimA- | animal |
| UPEC | IMT15013 | 73 | 73 | B2 | GimA- | animal |
| UPEC | IMT14953 | 925 | None | D | GimA- | animal |
| UPEC | IMT15020 | 88 | 23 | B1 | GimA- | animal |
| UPEC | IMT15027 | 937 | None | AxB1 | GimA- | animal |
| Human_fecal_ | ECOR-01 | 10 | 10 | A | GimA- | human |
| Human_fecal_ | ECOR-02 | 49 | 10 | A | GimA- | human |
| Animal_fecal_ | ECOR-03 | 10 | 10 | A | GimA- | animal |
| Human_fecal_ | ECOR-04 | 63 | None | A | GimA- | human |
| Human_fecal_ | ECOR-05 | 10 | 10 | A | GimA- | human |
| Human_fecal_ | ECOR-06 | 77 | 206 | AxB1 | GimA- | human |
| Animal_fecal_ | ECOR-07 | 87 | None | AxB1 | GimA- | animal |
| Human_fecal_ | ECOR-08 | 10 | 10 | A | GimA- | human |
| Human_fecal_ | ECOR-09 | 10 | 10 | A | GimA- | human |
| Human_fecal_ | ECOR-10 | 43 | 10 | A | GimA- | human |
| UPEC | ECOR-11 | 10 | 10 | A | GimA- | human |
| Human_fecal_ | ECOR-12 | 10 | 10 | A | GimA- | human |
| Human_fecal_ | ECOR-13 | 44 | 10 | A | GimA- | human |
| UPEC | ECOR-14 | 10 | 10 | A | GimA- | human |
| Human_fecal_ | ECOR-15 | 45 | None | AxB1 | GimA- | human |
| Animal_fecal_ | ECOR-16 | 46 | 46 | AxB1 | GimA- | animal |
| Animal_fecal_ | ECOR-17 | 47 | None | AxB1 | GimA- | animal |
| Animal_fecal_ | ECOR-18 | 48 | 10 | A | GimA- | animal |
| Animal_fecal_ | ECOR-19 | 48 | 10 | A | GimA- | animal |
| Animal_fecal_ | ECOR-20 | 48 | 10 | A | GimA- | animal |
| Animal_fecal_ | ECOR-21 | 48 | 10 | A | GimA- | animal |
| Animal_fecal_ | ECOR-22 | 50 | None | A | GimA- | animal |
| Animal_fecal_ | ECOR-23 | 51 | None | ABD | GimA- | animal |
| Human_fecal_ | ECOR-24 | 52 | None | AxB1 | GimA- | human |
| Animal_fecal_ | ECOR-25 | 10 | 10 | A | GimA- | animal |
| Human_fecal_ | ECOR-26 | 53 | None | AxB1 | GimA- | human |
| Animal_fecal_ | ECOR-27 | 53 | None | AxB1 | GimA- | animal |
| Human_fecal_ | ECOR-28 | 54 | None | AxB1 | GimA- | human |
| Animal_fecal_ | ECOR-29 | 55 | 155 | AxB1 | GimA- | animal |
| Animal_fecal_ | ECOR-30 | 56 | 155 | ABD | GimA- | animal |
| Animal_fecal_ | ECOR-31 | 57 | 350 | ABD | GimA- | animal |
| Animal_fecal_ | ECOR-32 | 56 | 155 | ABD | GimA- | animal |
| Animal_fecal_ | ECOR-33 | 56 | 155 | ABD | GimA- | animal |
| Animal_fecal_ | ECOR-34 | 58 | 155 | AxB1 | GimA- | animal |
| Human_fecal_ | ECOR-35 | 59 | 59 | ABD | GimA- | human |
| Human_fecal_ | ECOR-36 | 60 | None | ABD | GimA- | human |
| Animal_fecal_ | ECOR-37 | 61 | 11 | D | GimA- | animal |
| Human_fecal_ | ECOR-38 | 62 | None | ABD | GimA- | human |
| Human_fecal_ | ECOR-39 | 62 | None | ABD | GimA- | human |
| UPEC | ECOR-40 | 62 | None | ABD | GimA- | human |
| Human_fecal_ | ECOR-41 | 62 | None | ABD | GimA- | human |
| Human_fecal_ | ECOR-42 | 64 | None | ABD | GimA- | human |
| Human_fecal_ | ECOR-43 | 65 | None | ABD | GimA- | human |
| Animal_fecal_ | ECOR-44 | 66 | None | D | GimA- | animal |
| Animal_fecal_ | ECOR-45 | 67 | None | AxB1 | GimA- | animal |
| Animal_fecal_ | ECOR-46 | 68 | None | D | GimA- | animal |
| Animal_fecal_ | ECOR-47 | 69 | 69 | D | GimA- | animal |
| Human_fecal_ | ECOR-48 | 70 | None | D | GimA- | human |
| Human_fecal_ | ECOR-49 | 71 | None | D | GimA- | human |
| UPEC | ECOR-50 | 72 | 405 | D | GimA- | human |
| Human_fecal_ | ECOR-53 | 12 | 12 | B2 | GimA- | human |
| Human_fecal_ | ECOR-56 | 73 | 73 | B2 | GimA- | human |
| Animal_fecal_ | ECOR-58 | 75 | None | AxB1 | GimA- | animal |
| Human_fecal_ | ECOR-61 | 78 | None | B2 | GimA- | human |
| UPEC | ECOR-62 | 79 | None | B2 | GimA- | human |
| UPEC | ECOR-64 | 81 | 14 | B2 | GimA- | human |
| Animal_fecal_ | ECOR-67 | 84 | None | AxB1 | GimA- | animal |
| Animal_fecal_ | ECOR-68 | 85 | None | B1 | GimA- | animal |
| Animal_fecal_ | ECOR-69 | 86 | 86 | ABD | GimA- | animal |
| Animal_fecal_ | ECOR-70 | 88 | 23 | B1 | GimA- | animal |
| UPEC | ECOR-71 | 88 | 23 | B1 | GimA- | human |
| UPEC | ECOR-72 | 89 | None | AxB1 | GimA- | human |
